# A Case of Opium Narcosis, &c.

**Published:** 1889-05

**Authors:** T. H. Williams


### A CASE OF OPIUM NARCOSIS, WITH A DISCUSSION OF ITS TREATMENT.

BY T. H. WILLIAMS, M. D.,

Not long since I was solicited to see Mrs. T., who had taken 25 grains of morphiæ sulph. She had been under observation for some months for an anæmic condition of the brain, with spinal irritation. While in a state of melancholy she attempted a *felo de se* with a more potent agent that a mere bodkin. The morphiæ sulph. was ingested at 1 P. M. I saw her at 7 P. M.; the pupil was pinholed, the gait titubating and a disposition to dose. The respiration was easy, the pulse 95, slightly accelerated. An emesis of mustard water was used while the husband was dispatched for the necessary antagonistic agents. At 8 o'clock, with the condition unchanged, $\frac{1}{10}$ gr. of atropia was thrown under the skin of the forearm; at 8.30 the pupil was unaffected, and the dose was renewed. At 9 o'clock the pupil dilated slightly, but a new train of symptoms supervened, somnolence set in, respiration grew rapidly worse—its very autonomy menaced. I will here state, up to this time her mind was tranquil, she taking part in every effort to save herself, and would frequently interrogate me as to the possibility of her recovery.

To save the respiratory centres, $\frac{1}{20}$ gr. strychniæ sulph. was inserted under the skin of the forearm, but without avail, for in half an hour their function ceased. The pulse grew so rapid and so intermittent that it could not be counted. Artificial respiration was practiced and amyl nitrite employed for one hour, at the end of which the induced current was used; the anode placed at the dependent root of the phrenic nerve trunk, the cathode below the ensiform cartilage to the right of the median line: at this point a respiratory impulse could be produced at will. At 11 o'clock the atropia was renewed, the faradic current steadily maintained. The pulse fell to 120, the respiration rose to 10 per minute. At 12 o'clock the condition was also as above stated, $\frac{1}{20}$ gr. dose of strych. sulph. was renewed. At 4 A. M. there was as yet no substantial improvement, the race of the antagonism was unfinished and the results uncertain. A $\frac{1}{10}$ gr. atropia sulph. was renewed, and in less than one hour the prospects brightened, the lividity of the surface gave place to a more healthful appearance, the heating body gave evidence that this was a percursory of the blood returning to its accustomed paths. At 8 o'clock the antagonism succeeded and the patient awoke from her opium narcosis.

Eight hours transpired from the injection of this drug until its lethal effects were manifest. Thus did this agent secrete itself in the chylopoietic system for this duration before it began its work of death. So rapid was its effect that a half hour sufficed to demolish the respiratory centres. That there is an antagonism between opium and atropia there is not a doubt, and there is not a medicinal agent yet known whose antagonizing powers supersede this agent. Its power to stimulate the dominating centre of respiration cannot be controverted. When apnœa occurs from narcosis, when certain embarrassments, or failure, of the respiration ensue, whereby the autonomy of this special function is deranged, so that æration of the blood is arrested and its products of waste remain, the autonomy of this centre must be preserved until the system has disposed of the noxious element by its common sewerage, the excretory and eliminating organs.

Strychnia has been strongly urged and advocated, but a glance at the physiological action of this drug does not yield the anticipated result. The dominion of this motor excitant is confined to the spinal cord, and evidence is materially in want of its effects upon the central organ of the nervous system. The dominating centre of respiration, while residing in the *nœud vital* of the medulla oblongata, receives strands of nerve matter as a contribution from the cranial as well as the spinal system; and, inasmuch as this dominating centre owes its autonom to the cranial as well as the spinal nervous system, it must of necessity follow that an agent whose effects are limited to the spinal system cannot exercise complete inhibition over this centre.

Electricity is an agent of indubitable value. When the autonomy of the respiratory centres is intact and their responsive sensibility obtuned, the induced current is of inestimable value. By repeated demand upon the working capacity of this centre, exhaustion can be readily induced. The normal respiration should be imitated as far as possible and the connection broken at the instant the respiratory effort has begun, and replaced after the lapse of a few seconds; in this manner the apparatus can be used for an indefinite time without injury.

The heart's action in all apnoic narcotism is greatly influenced. Aside from the presence of blood gases and their effects on the inhibiting nerve, there is an intimate relation existing between this nerve root and the dominating centre of respiration. To preserve the autonomy of the circulating organ under such conditions is of the utmost importance. Cardiac stimuli are required; best of all is amyl nitrite, on account of its efficiency and easy mode of exhibition. The old system, the therapeutical mastigosis which decked the thirteenth and fourteenth century, should be, and it ought to be, relegated to the eternal past. It matters not however skilled the attendant may be, it matters not how firmly his faith is fastened to the doctrines of antagonism, he belies his faith when he requires or forces his patient to perform ambulatory tasks, or agonizes him with merciless flagellations, and startles him with deafening shrieks—mercy alone should plead for a rest from such treatment. Who has not seen a patient lost, who, after a legitimate antagonism, had succumbed from sheer exhaustion. Place the patient in bed with suitable covering, keep up the body temperature, lower the head, and do not molest or worry him beyond the necessity of enforcing antagonizing efforts.—*Col. and Clin. Record.*

